# Cervical Ripening with Low-Dose Prostaglandins in Planned Vaginal Birth after Cesarean

**DOI:** 10.1371/journal.pone.0080903

**Published:** 2013-11-19

**Authors:** Thomas Schmitz, Anne-Gaelle Pourcelot, Constance Moutafoff, Valérie Biran, Olivier Sibony, Jean-François Oury

**Affiliations:** 1 Service de Gynécologie Obstétrique, Hôpital Robert Debré, AP-HP, Paris, France; 2 Université Paris Diderot, Paris, France; 3 Service de Néonatalogie, Hôpital Robert Debré, AP-HP, Paris, France; Baylor College of Medicine, United States of America

## Abstract

**Objectives:**

To compare uterine rupture, maternal and perinatal morbidity rates in women with one single previous cesarean after spontaneous onset of labor or low-dose prostaglandin-induced cervical ripening for unfavourable cervix.

**Study Design:**

This was a retrospective cohort study of 4,137 women with one single previous cesarean over a 22-year period. Inpatient prostaglandin administration consisted in single daily local applications.

**Results:**

Vaginal delivery was planned for 3,544 (85.7%) patients, 2,704 (76.3%) of whom delivered vaginally (vaginal birth after Cesarean (VBAC) rate = 65.4%). Among women receiving prostaglandins (n=515), 323 (62.7%) delivered vaginally. Uterine rupture (0.7% compared with 0.8%, OR 1.1, 95% CI 0.4-3.4, p=0.88), maternal (0.9% compared with 1.2%, OR 1.3, 95% CI 0.5-3.2, p=0.63) and perinatal (0.3% compared with 0.8%, OR 2.4, 95% CI 0.7-8.5, p=0.18) morbidity rates did not differ significantly between patients with spontaneous onset of labor and those receiving prostaglandins, nor did these rates differ according to the planned mode of delivery.

**Conclusion:**

In comparison with patients with spontaneous labor, inducing cervical ripening with low-dose prostaglandins in case of unfavourable cervix is not associated with appreciable increase in uterine rupture, maternal or perinatal morbidity.

## Introduction

Planned vaginal delivery in women with one single previous cesarean is associated with both short- and long-term benefits and risks for mothers and neonates. In comparison with planned cesarean, planned vaginal delivery is associated with reduced maternal mortality but increased rates of uterine rupture, subsequent hypoxic ischemic encephalopathy and perinatal death [1]. Rates and relative risks for these events are low. It has been calculated that in a hypothetical group of 100,000 patients at term gestational age, planning vaginal birth after cesarean (VBAC) rather than cesarean would result in 10 fewer maternal deaths, 650 additional uterine ruptures, and 50 additional neonatal deaths [1]. Therefore, planned vaginal delivery seems a reasonable option for most women with a single previous cesarean delivery [1], and offering this option to them, in the absence of contraindication, is encouraged by the American, Canadian, and French Colleges of Obstetricians and Gynecologists [2-4]. 

After two cesareans, women will generally deliver by cesarean in all their future pregnancies. The long-term effects of repeated cesareans are only now being elucidated. Rates of placenta previa, placenta accreta, transfusion, surgical injury, and hysterectomy are all strongly correlated with the increasing number of prior cesareans [5-7]. Mathematical models integrating these risks and maternal desire for future pregnancies have concluded that planning VBAC for the second delivery, compared with elective repeat cesarean delivery, should result in fewer hysterectomies in future pregnancies [8,9]. Therefore, the benefits expected from a planned VBAC might be greater for patients who want more children.

Although VBAC rates have dropped over time, rates of successful trial of labor have remained constant, around 75% [1]. Accordingly, to increase VBAC rates and decrease the complications related to multiple cesareans, rates of planned cesarean should fall and rates of planned vaginal delivery should increase. One step towards achieving these goals might be the planning of vaginal delivery even in cases of unfavourable cervix, when a fetal or maternal condition indicates delivery. In these situations, planned cesarean is often performed because obstetricians might be reluctant to use prostaglandins to induce cervical ripening in view of the several case series and large cohort studies that have reported an association between their use and increased rates of uterine rupture [10-14]. The protocols of prostaglandin administration in these studies, however, were either not specified or consisted in high and/or closely repeated prostaglandin doses, regimens that might account for the increased rates of complications that have been reported.

The aim of this retrospective study of a large cohort of women with one single previous cesarean was therefore to compare uterine rupture and maternal and perinatal morbidity rates after low-dose prostaglandin-induced cervical ripening or spontaneous onset of labor.

## Materials and Methods

This study examined the records of the women with one single previous cesarean delivered in our university-hospital level III maternity unit (3,000 deliveries per year) from May 2, 1988, through December 31, 2010. We included all women with one single previous cesarean delivering at or after 37 weeks of gestation and excluded only those women with non cephalic presentations, multi-fetal pregnancies and prior classical uterine incision. Consequently, women with pregnancy complications such as hypertension, preeclampsia, diabetes, intrauterine growth restriction were not excluded from the study.

**Figure 1:**
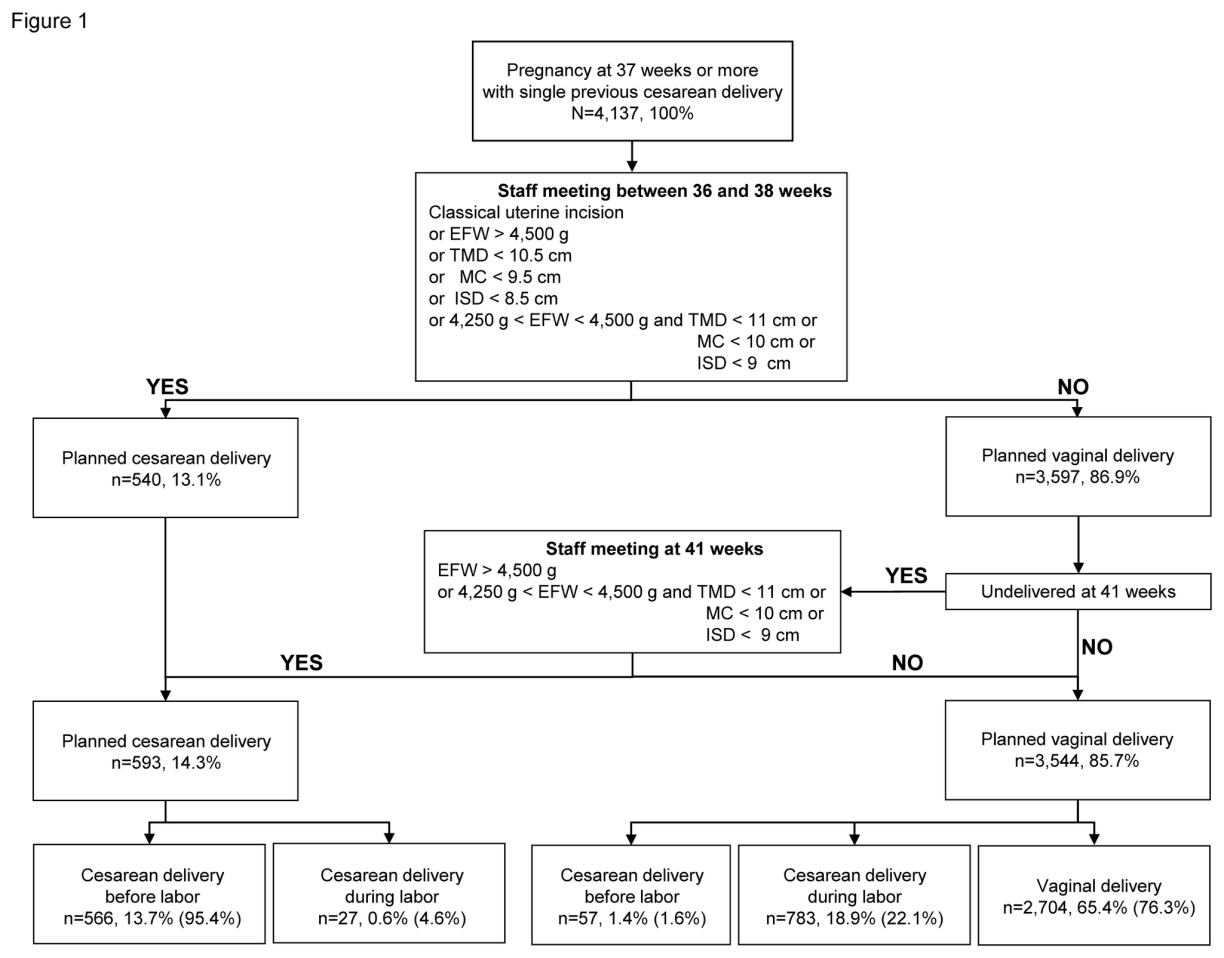
Decision tree for the planned mode of delivery and effective mode of delivery of the study population. Percentages in parenthesis are calculated for the planned mode of delivery, percentages not in parenthesis are calculated for the total population. EFW: estimated fetal weight. TMD: transverse median diameter. MC: median conjugate. ISD: intraspinous diameter.

In our institution, route of delivery is decided collectively at the daily obstetric staff meeting between 36 and 38 weeks of gestation and then explained and discussed with patients. If the patient remains undelivered, route of delivery is discussed again at 41 weeks of gestation. Cesarean is planned in cases of i) prior classical uterine incision, ii) highly abnormal pelvic measurement (transverse median diameter (TMD) less than 10.5 cm or median conjugate (MC) less than 9.5 cm or interspinous diameter (ISD) less than 8.5 cm) at the computed tomographic (CT) pelvimetry performed between 35 and 37 weeks, iii) ultrasound estimated fetal weight (EFW) between 4,250 and 4,500g and TMD less than 11 cm or MC less than 10 cm or ISD less than 9 cm, and iv) ultrasound EFW above 4,500 g whatever the pelvic measurements. This EFW threshold has been chosen because we consider that rates of successful trial of labor are too low and rates of uterine rupture too high in case of an EFW more than 4,500g, as suggested by Elkousy et al. [15]. In these cases, planned cesareans are performed at 39 weeks' gestation. For fetus with EFW between 4,250g and 4,500 g at 41 weeks, the planned route of delivery is discussed according to other risk factors of failed trial of labor and uterine rupture, such as prior cesarean for dystocia if available or absence of previous vaginal delivery, on a case by case basis. In all other clinical situations, vaginal delivery is proposed. The decision tree for the planned mode delivery is summarized in Figure 1.

Only if a medical condition indicates delivery, induction by oxytocin is authorized when the Bishop score is 6 or more. If not, cervical ripening is induced with prostaglandins, before labor induction with oxytocin if the Bishop score is 6 or more after prostaglandin applications.. All women treated with prostaglandins in this study had therefore a Bishop score less than 6. The protocol consists in single daily inpatient prostaglandin applications for up to 7 days with 0.5 mg intracervical dinosprostone until 1993. Since 1993, women are treated with 0.5 mg intracervical dinosprostone administered on Days 1, 2, and 3, and misoprostol (25, 50, 50 and 50 μg, respectively) placed in the posterior fornix on Days 4, 5, 6 and 7 respectively. Patients are observed in the labor ward with continuous fetal monitoring during the 2 hours following prostaglandin treatment. In the absence of painful uterine contraction and in case of Bishop score less than 6, patients are allowed to get back to their room till the next day and fetal heart rate and uterine contractions are monitored in the evening. Day 7, if the Bishop score remains less than 6, 4 hours after misoprostol application, labor induction with oxytocin infusion is not attempted and a cesarean before labor is performed. During labor, oxytocin infusion is indicated only after arrest of dilatation with uterine hypotonic dysfunction that affects either the frequency or intensity of contractions. After two hours of failure to progress under oxytocin infusion, a cesarean delivery is performed.

Maternal, obstetric and early neonatal data are collected prospectively daily and recorded in our computerized database by midwives during hospitalization and immediately after delivery. Data entered into the database are double-checked every morning for each delivery by the obstetrician in charge of the daily staff meeting and by the midwife dedicated to the database maintenance. Details of maternal age, parity, previous vaginal delivery, pregnancy complications, labor induction, mode of delivery, gestational age at delivery, and birth weight were reviewed. Uterine rupture was defined as complete disruption of the uterine muscle and visceral peritoneum. Uterine dehiscence was defined as a disruption of the uterine muscle with intact serosa. Maternal morbidity was defined by a composite measure including uterine rupture, bladder injury, surgery (compression sutures, artery ligation, and hysterectomy) for postpartum hemorrhage, transfer to the intensive care unit, or death. 

Perinatal data were extracted from our computerized database and crossmatched with the neonatal intensive care unit database. The clinical notes from the paper files of all neonates with 5-minute Apgar score less than 4, umbilical cord arterial pH less than 7.0 or born after uterine rupture were carefully examined. Perinatal morbidity was defined as perinatal asphyxia or antepartum stillbirth not related to multiple-malformation syndrome after 39 weeks of gestation. Perinatal asphyxia was defined as a 5-minute Apgar score less than 4 or umbilical cord arterial pH less than 7.0.

The primary outcome was uterine rupture. The secondary outcomes were maternal and perinatal morbidity. We first compared maternal and perinatal characteristics and the primary and secondary outcomes according to the planned mode of delivery and then to labor status, with Pearson’s χ^2^ test or Fisher's exact test when appropriate. We then estimated the odds ratios and their 95% confidence intervals for the primary and secondary outcomes with the spontaneous onset of labor group as the reference. One-way analysis of variance and *t* tests were used for quantitative variables. Statistical significance was defined by a p value less than 0.05. We used Stata 9.2 software (StataCorp LP, College Station, TX).

Ethics statement: The institutional review board, “Comité de Protection des Personnes” Paris Ile-de-France III, has examined this work, found it conformed to the ethical standards and to the scientific requirements applicable to medical research and waived the need for informed consent of the participants because of the retrospective nature of the study over a 22-year period. 

## Results

During the 22-year study period, 4,137 women with one single previous cesarean gave birth at or after 37 weeks of gestation in our level III maternity facility. Cesarean was planned for 593 (14.3%) women mainly because of suspected cephalopelvic disproportion (n=533). Vaginal birth was planned for 3,544 (85.7%) patients. Among the planned vaginal deliveries, 57 (1.6%) patients had cesarean before labor because prostaglandin-induced cervical ripening failed, 783 (22.1%) cesarean during labor because of arrest of dilatation (n=465), abnormal fetal heart rate (n=293), maternal indications (n=12), and suspected uterine rupture (n=17), and 2,704 (76.3%) delivered vaginally. The VBAC rate was thus 65.4% (Figure 1).

As shown in Table 1, patients with planned cesareans had fewer previous vaginal deliveries, more pregnancy complications, and gave birth at significantly earlier gestational ages than women in the planned vaginal delivery group.

**Table 1:** Maternal and neonatal characteristics according to the planned mode of delivery (N=4,137).

		Planned cesarean (n=593)	Planned vaginal (n=3,544)	p
Maternal age	Mean (y)	32.0±4.7	32.7±4.9	<0.01
	< 21 y	5 (0.8)	24 (0.7)	
	21-38 y	493 (83.2)	2,938 (82.9)	
	> 38 y	95 (16.0)	582 (16.4)	0.88
Geographical origin	Europe	349 (58.9)	1,957 (55.2)	
	Central and West Africa	139 (23.4)	709 (20.1)	
	North africa	76 (12.8)	663 (18.7)	
	South East Asia	18 (3.0)	161 (4.5)	
	India	11 (1.9)	54 (1.5)	<0.01
Previous vaginal delivery		115 (19.4)	1,385 (39.1)	<0.01
Labor induction		0 (0.0)	766 (21.6)	-
No pregnancy complication		506 (85.3)	3,216 (90.7)	<0.01
Pregnancy complications	Hypertension, preeclampsia	28 (4.7)	159 (4.5)	0.80
	Diabetes	47 (7.9)	99 (2.8)	<0.01
	Thromboembolism	0 (0.0)	6 (0.2)	0.39
	Intrauterine growth restriction	5 (0.8)	57 (1.6)	0.16
	Placenta previa	13 (2.2)	33 (0.9)	<0.01
	Hypertension, preeclampsia			
Gestational age	Mean (wk)	39.4±1.0	39.8±1.2	<0.01
	> 41 wk	53 (7.1)	696 (19.6)	<0.01
Birth weight	Mean (g)	3,405±502	3,408±472	0.93
	< 2,500 g or less	22 (3.7)	89 (2.5)	
	2,500-4,000 g	502 (84.7)	3,081 (86.9)	
	> 4,000 g	69 (11.6)	374 (10.6)	0.17

Data are n (%) or mean±standard deviation

Uterine rupture was diagnosed in 3 (0.5%) women in the planned cesarean delivery group and in 31 (0.9%) in the planned vaginal delivery group (p=0.36). Uterine dehiscence was more frequent after planned vaginal than planned cesarean delivery (1.6% compared with 0.5%, p=0.04) (Table 2). Maternal morbidity did not differ significantly between the planned cesarean and planned vaginal delivery groups (0.8% compared with 1.2%, p=0.44).

**Table 2:** Maternal and perinatal morbidity according to the planned mode of delivery (N=4,137).

	Planned cesarean (n=593)	Planned vaginal (n=3,544)	p
**Maternal**			
Uterine rupture	3 (0.5)	31 (0.9)	0.36
Uterine dehiscence	3 (0.5)	58 (1.6)	0.04
Bladder injury	0 (0.0)	7 (0.2)	0.60
Prostaglandins for post-partum hemorrhage	21 (3.5)	120 (3.4)	0.85
Surgery for post-partum hemorrhage	0 (0.0)	3 (0.08)	1.00
Transfer to intensive care unit	2 (0.3)	5 (0.1)	0.27
Death	0 (0.0)	0 (0.0)	NA
Maternal morbidity*	5 (0.8)	43 (1.2)	0.44
**Perinatal**			
5’ Apgar score < 4	0 (0.0)	13 (0.4)	0.24
Umbilical cord arterial pH < 7.0**	3 (0.5)	10 (0.3)	0.37
Intubation in delivery room	7 (1.2)	29 (0.8)	0.38
Transfer to neonatalogy unit	58 (9.8)	198 (5.6)	<0.01
Transfer to neonatal intensive care unit	9 (1.5)	34 (1.0)	0.22
Perinatal asphyxia***	3 (0.5)	15 (0.4)	0.78
Antepartum stillbirth	0 (0.0)	11 (0.3)	0.38
Antepartum stillbirth without polymalformation syndrome > 39 wk	0 (0.0)	2 (0.06)	1.00
Intrapartum stillbirth	0 (0.0)	0 (0.0)	NA
Neonatal death	0 (0.0)	7 (0.2)	0.37
Neonatal death due to perinatal asphyxia	0 (0.0)	3 (0.08)	1.00
Perinatal morbidity****	3 (0.5)	17 (0.5)	0.93

Data are n (%). NA: not applicable.

* Uterine rupture, bladder injury, surgery for postpartum hemorrhage, transfer to intensive care unit or death

** Umbilical cord arterial pH was available for 3,331 neonates (80.5%)

*** Apgar score at 5 minutes less than 4 or pH less than 7.0

**** Perinatal asphyxia, neonatal death due to perinatal asphyxia or antepartum stillbirth without polymalformation after 39 weeks

Perinatal morbidity did not differ significantly between the planned cesarean and vaginal delivery groups (0.5% compared with 0.5%, p=0.93). In the planned vaginal delivery group, 15 (0.4%) neonates experienced perinatal asphyxia (Table 2). Three of these neonates died in NICU, one after uterine rupture. The other 12 neonates recovered without sequela. The other four neonatal deaths in the planned vaginal delivery group were all related to polymalformation syndromes. Of the 11 prepartum stillbirths that prompted planned vaginal delivery, only two occurred after 39 weeks of gestation and without a polymalformation syndrome.

In the planned vaginal delivery group, prostaglandins were administered to 515 patients with unfavourable cervix (14.5%) because of fetal indications (n=239), maternal indications (n=124), premature rupture of membranes (n=99), antenatal stillbirth (n=5), and unknown reasons (n=48) and 251 women with favourable cervix had labor induced with oxytocin. Patients receiving prostaglandins had fewer previous vaginal deliveries (32.4% compared to 42.4%, p<0.01), more pregnancy complications (24.3% compared to 5.6%, p<0.01), gave birth at significantly more advanced gestational ages (39.9 ± 1.4 compared to 39.7 ± 1.1, p<0.01) and had smaller neonates (3,309 ± 550 compared to 3,370 ± 444, p<0.01) than women with spontaneous labor. As shown in Table 3, the highest rate of vaginal delivery (81.1%) was observed after spontaneous onset of labor when no oxytocin infusion was required for labor augmentation. The lowest rates of vaginal delivery were associated with prostaglandins used to induce cervical ripening (60.2% to 65.6%). The rate of vaginal delivery was significantly higher among women with spontaneous onset of labor than in any of the other groups. Uterine rupture and maternal and perinatal morbidity rates did not differ significantly according to labor status (Table 3). No neonatal death related to perinatal asphyxia occurred in the group of patients receiving prostaglandins. 

**Table 3:** Maternal and perinatal outcomes according to labor status among women with planned vaginal delivery (N=3,544).

	Vaginal delivery			Uterine rupture			Maternal morbidity*			Perinatal morbidity**		
Type of labor	n (%)	OR (95% CI)	p	n (%)	OR (95% CI)	p	n (%)	OR (95% CI)	p	n (%)	OR (95% CI)	p
Spontaneous n=1,828 (51.6)	1,483 (81.1)	Ref		13 (0.7)	Ref		17 (0.9)	Ref		6 (0.3)	Ref	
Induction or augmentation n=1,716 (48.4)	1,221 (71.2)	0.69 [0.49-0.67]	<0.01	18 (1.0)	1.5 [0.7-3.0]	0.28	26 (1.5)	1.6 [0.9-3.0]	0.11	9 (0.5)	1.6 [0.6-4.5]	0.37
Prostaglandins n=515 (14.5)	323 (62.7)	0.39 [0.32-0.48]	<0.01	4 (0.8)	1.1 [0.4-3.4]	0.88	6 (1.2)	1.3 [0.5-3.2]	0.63	4 (0.8)	2.4 [0.7-8.5]	0.18
Oxytocin (only) n=1,201 (33.9)	898 (74.8)	0.69 [0.58-0.82]	<0.01	14 (1.2)	1.6 [0.8-3.5]	0.20	20 (1.7)	1.8 [0.9-3.5]	0.08	5 (0.4)	1.3 [0.4-4.2]	0.70
Prostaglandins (only) n=274 (7.7)	165 (60.2)	0.35 [0.27-0.46]	<0.01	2 (0.7)	1.0 [0.2-4.5]	0.97	3 (1.1)	1.2 [0.3-4.0]	0.79	2 (0.7)	2.2 [0.4-11.1]	0.33
Prostaglandins + Oxytocin n=241 (6.8)	158 (65.6)	0.44 [0.33-0.59]	<0.01	2 (0.8)	1.2 [0.3-5.2]	0.84	3 (1.2)	1.3 [0.4-4.6]	0.64	2 (0.8)	2.5 [0.5-12.7]	0.25
Oxytocin (induction) n=251 (7.1)	193 (76.9)	0.77 [0.56-1.06]	0.14	3 (1.2)	1.7 [0.5-6.0]	0.41	4 (1.6)	1.7 [0.6-5.2]	0.33	0 (0.0)	NA	
Oxytocin (augmentation) n=950 (26.8)	705 (74.2)	0.67 [0.56-0.81]	<0.01	11 (1.2)	1.6 [0.7-3.7]	0.23	16 (1.7)	1.8 [0.9-3.6]	0.09	5 (0.5)	1.6 [0.5-5.3]	0.44
Total N=3,544 (100)	2,704 (76.3)			31 (0.9)			43 (1.2)			15 (0.4)		

* Uterine rupture, bladder injury, surgery for postpartum hemorrhage, transfer to intensive care unit or death

** Perinatal asphyxia or neonatal death due to perinatal asphyxia

Finally, the rate of cesarean deliveries before labor was positively, and the vaginal delivery rate negatively, correlated with the increasing number of prostaglandin applications (Table 4). The four cases of uterine rupture associated with prostaglandin use all occurred after the first application. None of these cases was associated with perinatal asphyxia or neonatal death.

**Table 4:** Mode of delivery according to the number of prostaglandin applications (N=515).

Prostaglandin applications	Cesarean before labor	p*	Cesarean during labor	p*	Vaginal delivery**	p*	Vaginal delivery***	p*
One, n=285 (55.3)	21 (7.4)		66 (23.1)		198 (69.5)		198 (75.0)	
Two, n=117 (22.7)	10 (8.5)		40 (34.2)		67 (57.3)		67 (62.6)	
Three, n=52 (10.1)	8 (15.4)		14 (26.9)		30 (57.8)		30 (68.2)	
Four and more, n=61 (11.9)	18 (29.5)	<0.01	15 (24.6)	0.42	28 (45.9)	<0.01	28 (65.1)	0.96
Total, N=515 (100)	57 (11.1)		135 (26.2)		323 (62.7)		323 (70.5)	

Data are n (%).

* for trend

** total deliveries as denominator

*** Cesareans during labor plus vaginal deliveries as denominator

## Discussion

Our study shows that it is possible to achieve high rates of vaginal delivery in women with a single previous cesarean without simultaneously increasing rates of uterine rupture and maternal and perinatal morbidity.

We report herein a 65.4% VBAC rate. This rate is much higher than usually reported in US (ranging from 32% [16] to 37% [17, 18]), Australian (20%) [14] or Dutch (54%) [13] studies; it is also higher than the French (36% in 2010) [19] and US (8.7% in 2007) [1] national rates. It is higher mainly because VBAC was attempted in 85.7% of our patients, compared with trial of labor rates ranging from 38% to 72% in Australian, US and Dutch studies [13,14,16-18]. Planned vaginal delivery was successful in 76.3% of our cases, consistent with the rates reported in the literature [13,14,16-18]. Our study thus demonstrates, yet again, that VBAC rates are strongly correlated with planned vaginal delivery rates. 

One major reason that might explain our high VBAC rate is that 14.5% of the women with planned vaginal deliveries were treated with prostaglandins to induce cervical ripening, and almost two-thirds of them delivered vaginally. This rate of prostaglandin use in patients with attempted VBAC is two to five times higher than those reported in the US (ranging from 2.8% to 8.1%) [10-12,20,21], Australian (7.4%) (14) or Dutch (6.4%) [13] studies. 

This high VBAC rate was not associated with either a significant increase in uterine rupture rates or increased maternal or perinatal morbidity, neither did these rates increased in the prostaglandin group in comparison with the spontaneous labor group. The rates we report herein of uterine rupture and neonatal death due to perinatal asphyxia, by planned mode of delivery and labor status, are consistent with the international literature [1,10-14,16-18,20]. It is unlikely that the differences evidenced in the baseline characteristics between the spontaneous labor and prostaglandin groups could explain the absence of increased risks for the primary and secondary outcomes in the prostaglandin group, since pregnancy complications and absence of previous vaginal delivery were more frequent in that group. Unfortunately, it was not possible to estimate adjusted odds ratios by logistic regression models because events in the prostaglandin group were too scarce. We believe that our low uterine rupture rate despite common use of prostaglandins is most likely related to the protocol for prostaglandin administration. 

Our protocol of prostaglandin administration takes into account the fact that the uterus is scarred. It consists of single daily inpatient low dose (0.5 mg) intracervical dinosprostone aimed at inducing cervical ripening but not labor at any cost. Previous small retrospective studies (30 and 117 patients) of the use of 0.5 mg intracervical dinoprostone reported no cases of uterine rupture [22,23]. The studies that have shown increased rates of uterine ruptures associated with prostaglandin use either did not specify their protocols of prostaglandin administration [12-14,20,21] or used higher doses than ours; their doses ranged from 2 mg [11] to 4 mg [10] and were repeated every 4 [10] to 6 hours [11]. It appears likely that the differences between our protocol of prostaglandin administration for women with previous cesarean and those described above [10,11] explain, at least in part, the discrepancies in uterine rupture rates. Use of misoprostol has been associated with increased rates of uterine rupture when VBAC is attempted [24,25]. Again, the doses used in these studies were higher and closer together than in our series. More importantly, we think, uterine reactivity to prostaglandins was not tested before misoprostol use by the serial applications of dinoprostone we normally use.

It is noteworthy that the rates of cesareans before labor for failed prostaglandin-induced cervical ripening in the planned vaginal delivery group increased with the number of prostaglandin applications. This finding confirms that we were opposed to induce labor with oxytocin when the cervix remained persistently unfavourable, in view of the potential association with increased uterine rupture rates. Finally, the rate of successful planned vaginal delivery in prostaglandin-treated women was above the 60% threshold below which major maternal morbidity and neonatal morbidity rates could be significantly increased [26].

In France and in our maternity, patients delivering by planned cesarean usually enter hospital the day before surgery and are sent back home on day 5 post partum, for a total length of hospitalization of 7 days, whereas women giving birth vaginally leave the maternity on day 3 postpartum, for a total length of 4 days. Therefore, the number of days of hospitalization in case of systematic planned cesarean for the 515 women with unfavourable cervix would have been 3,605 if such a strategy had been chosen whereas our inpatient cervical ripening policy resulted in 2,889 days of hospitalization. Therefore, although a more rigorous medico-economic evaluation might be required before drawing definitive conclusions, it is unlikely that we increased medical costs by offering inpatient cervical ripening to these patients.

Our study has several strengths and weaknesses, both related to its retrospective single-centre nature. We were able to provide detailed information regarding prostaglandin administration that is absent in large population-based or multicenter studies because our protocol was almost not modified during the 22-year study period. This information may be useful to obstetricians for counselling patients in this situation. We must acknowledge that our study lacked the statistical power to show significant differences in rare and severe adverse events related to vaginal delivery, such as neonatal death due to perinatal asphyxia. However, the fact that such morbid events occurred i) only three times in 3,544 consecutive planned vaginal deliveries over a 22-year period, and ii) in the range of the reported rates in large prospective studies [20] is reassuring regarding both the safety of our protocol and the external validity of our results. Furthermore, our study still had i) 80% power to detect a five fold increase of perinatal asphyxia rate from 0.3% to 1.5% and ii) 70% power to detect a three fold increase of uterine rupture rate from 0.7% to 2.1%, the rate reported in the literature [1] to be associated with the use of prostaglandins in comparison with spontaneous labor. Finally, use of CT pelvimetry remains controversial because of its potential association with increased rates of cesarean and risks of cancer in childhood. However, we and other have already shown that it could be informative and helpful for deciding the planned route of delivery [27,28] and our present results do not support at all an association between its use and increased rates of cesarean. Furthermore, the link between X-ray intrauterine exposure and leukemia in childhood suspected after publication of case-reports has not been confirmed by large cohort studies [29,30].

In conclusion, our results suggest that, for women with one single previous cesarean, planned vaginal delivery remains a reasonable option that can be associated with high vaginal delivery rates. Instead of planning a cesarean, low-dose prostaglandins to induce cervical ripening in case of unfavourable cervix could be offered more often to women with one single previous cesarean and thus ultimately decrease the maternal morbidity associated with repeated cesareans.
